# (±)-2-Methyl­piperazin-1-ium perchlorate

**DOI:** 10.1107/S160053681002862X

**Published:** 2010-07-24

**Authors:** Cong-Hu Peng

**Affiliations:** a Department of Chemical and Environmental Engineering, Anyang Institute of Technology, Anyang 455000, People’s Republic of China

## Abstract

In the title compound, C_5_H_13_N_2_^+^·ClO_4_^−^, the monoprotonated piperazine ring adopts a chair conformation. In the crystal structure, cations and anions are linked by inter­molecular N—H⋯O and N—H⋯N hydrogen bonds into layers parallel to (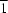
01).

## Related literature

For the properties of simple mol­ecular–ionic crystals, see: Czupiński *et al.* (2002 ▶); Katrusiak & Szafrański (1999 ▶, 2006 ▶).
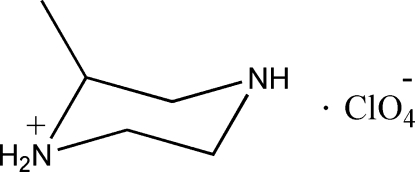


## Experimental

### 

#### Crystal data

C_5_H_13_N_2_^+^·ClO_4_^−^
*M* *_r_* = 200.62
Monoclinic, 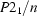

*a* = 6.8977 (5) Å
*b* = 8.1292 (6) Å
*c* = 16.2201 (11) Å
β = 98.614 (3)°
*V* = 899.25 (11) Å^3^
*Z* = 4
Mo *K*α radiation
μ = 0.41 mm^−1^
*T* = 293 K
0.30 × 0.25 × 0.20 mm

#### Data collection

Rigaku SCXmini diffractometer
Absorption correction: multi-scan (*CrystalClear*; Rigaku, 2005 ▶) *T*_min_ = 0.80, *T*_max_ = 0.90
8953 measured reflections
2055 independent reflections
1541 reflections with *I* > 2σ(*I*)
*R*_int_ = 0.040

#### Refinement

*R*[*F* ^2^ > 2σ(*F* ^2^)] = 0.075
*wR*(*F* ^2^) = 0.224
*S* = 1.05
2055 reflections
109 parameters
H-atom parameters constrained
Δρ_max_ = 0.86 e Å^−3^
Δρ_min_ = −0.56 e Å^−3^

### 

Data collection: *CrystalClear* (Rigaku, 2005 ▶); cell refinement: *CrystalClear*; data reduction: *CrystalClear*; program(s) used to solve structure: *SHELXS97* (Sheldrick, 2008 ▶); program(s) used to refine structure: *SHELXL97* (Sheldrick, 2008 ▶); molecular graphics: *SHELXTL* (Sheldrick, 2008 ▶); software used to prepare material for publication: *SHELXL97*.

## Supplementary Material

Crystal structure: contains datablocks I, global. DOI: 10.1107/S160053681002862X/rz2472sup1.cif

Structure factors: contains datablocks I. DOI: 10.1107/S160053681002862X/rz2472Isup2.hkl

Additional supplementary materials:  crystallographic information; 3D view; checkCIF report

## Figures and Tables

**Table 1:** Hydrogen-bond geometry (Å, °)

*D*—H⋯*A*	*D*—H	H⋯*A*	*D*⋯*A*	*D*—H⋯*A*
N1—H1*C*⋯O1	0.90	2.38	3.258 (6)	166
N1—H1*C*⋯O3	0.90	2.54	3.250 (5)	136
N2—H2*D*⋯O2^i^	0.90	2.43	2.998 (7)	121
N2—H2*C*⋯N1^ii^	0.90	1.99	2.883 (4)	169

Symmetry codes: (i) 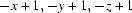; (ii) 
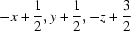
.

**Fig. 1.**
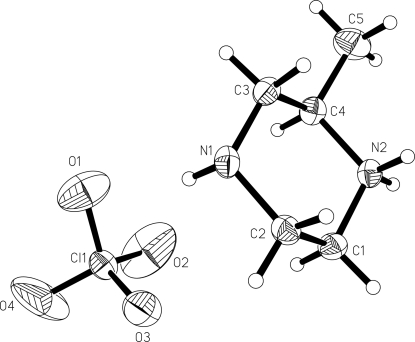
The asymmetric unit of the title compound with atom labels. Displacement ellipsoids were drawn at the 30% probability level

**Fig. 2.**
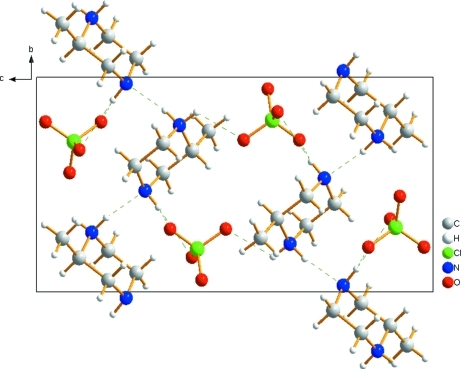
Packing viewed along the a axis. Hydrogen bonds are drawn as dashed lines

## supplementary crystallographic information

### Comment

Recently, much attention has been devoted to simple molecular–ionic crystals containing organic cations and acid radicals in 1:1 molar ratio due to the tunability of their special structural features and their interesting physical properties (Czupiński *et al.*, 2002; Katrusiak & Szafrański, 1999; Katrusiak & Szafrański, 2006). As a contribution in this field, the crystal structure of title salt is reported here.

The asymmetric unit of the title compound (Fig.1) consists of a monoprotonated 2-methylpiperazinium cation and a ClO_4_^-^anions. The piperazine ring adopts a chair conformation. In the crystal structure, cations and anions are linked by intermolecular N—H···O and N—H···N hydrogen bonds (Table 1) into layers parallel to the (1 0 1) plane (Fig.2).

### Experimental

(±)-2-Methylpiperazine (20 mmol) and 10% aqueous HClO_4_ solution in a molar ratio of 1:1 were mixed and dissolved in 25 ml water. The mixture was heated to 343 K to form a clear solution. On slow cooling of the reaction mixture to room temperature, block crystals of the title compound were formed.

### Refinement

All H atoms were placed in calculated positions, with C—H = 0.96–0.98Å and N—H = 0.90 Å, and refined using a riding model, with *U*_iso_(H) = 1.2*U*_eq_(C, N) or 1.5 *U*_eq_(C) for methyl H atoms.

### Crystal data

**Table:**

C_5_H_13_N_2_^+^·ClO_4_^−^	*F*(000) = 424
*M**_r_* = 200.62	*D*_x_ = 1.482 Mg m^−^^3^
Monoclinic, *P*2_1_/*n*	Mo *K*α radiation, λ = 0.71075 Å
Hall symbol: -P 2yn	Cell parameters from 1541 reflections
*a* = 6.8977 (5) Å	θ = 3.1–27.5°
*b* = 8.1292 (6) Å	µ = 0.41 mm^−^^1^
*c* = 16.2201 (11) Å	*T* = 293 K
β = 98.614 (3)°	Block, colourless
*V* = 899.25 (11) Å^3^	0.30 × 0.25 × 0.20 mm
*Z* = 4	

### Data collection

**Table:**

Rigaku SCXmini diffractometer	2055 independent reflections
Radiation source: fine-focus sealed tube	1541 reflections with *I* > 2σ(*I*)
graphite	*R*_int_ = 0.040
Detector resolution: 13.6612 pixels mm^-1^	θ_max_ = 27.5°, θ_min_ = 3.1°
ω scans	*h* = −8→8
Absorption correction: multi-scan (*CrystalClear*; Rigaku, 2005)	*k* = −10→10
*T*_min_ = 0.80, *T*_max_ = 0.90	*l* = −21→20
8953 measured reflections	

### Refinement

**Table:**

Refinement on *F*^2^	Primary atom site location: structure-invariant direct methods
Least-squares matrix: full	Secondary atom site location: difference Fourier map
*R*[*F*^2^ > 2σ(*F*^2^)] = 0.075	Hydrogen site location: inferred from neighbouring sites
*wR*(*F*^2^) = 0.224	H-atom parameters constrained
*S* = 1.05	*w* = 1/[σ^2^(*F*_o_^2^) + (0.1123*P*)^2^ + 1.4914*P*] where *P* = (*F*_o_^2^ + 2*F*_c_^2^)/3
2055 reflections	(Δ/σ)_max_ < 0.001
109 parameters	Δρ_max_ = 0.86 e Å^−^^3^
0 restraints	Δρ_min_ = −0.56 e Å^−^^3^

### Special details

**Table:**

Geometry. All e.s.d.'s (except the e.s.d. in the dihedral angle between two l.s. planes) are estimated using the full covariance matrix. The cell e.s.d.'s are taken into account individually in the estimation of e.s.d.'s in distances, angles and torsion angles; correlations between e.s.d.'s in cell parameters are only used when they are defined by crystal symmetry. An approximate (isotropic) treatment of cell e.s.d.'s is used for estimating e.s.d.'s involving l.s. planes.
Refinement. Refinement of *F*^2^ against ALL reflections. The weighted *R*-factor *wR* and goodness of fit *S* are based on *F*^2^, conventional *R*-factors *R* are based on *F*, with *F* set to zero for negative *F*^2^. The threshold expression of *F*^2^ > σ(*F*^2^) is used only for calculating *R*-factors(gt) *etc*. and is not relevant to the choice of reflections for refinement. *R*-factors based on *F*^2^ are statistically about twice as large as those based on *F*, and *R*- factors based on ALL data will be even larger.

### Fractional atomic coordinates and isotropic or equivalent isotropic displacement parameters (Å^2^)

**Table:**

	*x*	*y*	*z*	*U*_iso_*/*U*_eq_	
C1	0.4992 (5)	0.6921 (5)	0.6807 (2)	0.0414 (8)	
H1B	0.5975	0.7730	0.7019	0.050*	
H1A	0.5525	0.6233	0.6407	0.050*	
C2	0.4487 (5)	0.5875 (5)	0.7518 (2)	0.0406 (8)	
H2A	0.5644	0.5267	0.7759	0.049*	
H2B	0.4118	0.6591	0.7947	0.049*	
C3	0.1170 (5)	0.5531 (5)	0.6809 (2)	0.0389 (8)	
H3A	0.0599	0.6227	0.7194	0.047*	
H3B	0.0205	0.4703	0.6603	0.047*	
C4	0.1591 (5)	0.6579 (5)	0.6075 (2)	0.0384 (8)	
H4A	0.2062	0.5856	0.5664	0.046*	
C5	−0.0190 (7)	0.7490 (7)	0.5654 (3)	0.0646 (13)	
H5A	0.0152	0.8117	0.5196	0.097*	
H5B	−0.0656	0.8218	0.6048	0.097*	
H5C	−0.1201	0.6716	0.5452	0.097*	
Cl1	0.56264 (14)	0.19954 (12)	0.59443 (6)	0.0442 (4)	
N1	0.2890 (5)	0.4712 (4)	0.7261 (2)	0.0392 (7)	
H1C	0.3305	0.3941	0.6929	0.047*	
N2	0.3194 (4)	0.7764 (3)	0.63945 (18)	0.0352 (7)	
H2D	0.3494	0.8369	0.5966	0.042*	
H2C	0.2761	0.8453	0.6761	0.042*	
O1	0.3667 (7)	0.1613 (7)	0.6079 (3)	0.1041 (16)	
O2	0.5363 (11)	0.2923 (9)	0.5226 (3)	0.154 (3)	
O3	0.6614 (5)	0.2921 (5)	0.6628 (2)	0.0759 (11)	
O4	0.6557 (9)	0.0550 (9)	0.5835 (6)	0.215 (5)	

### Atomic displacement parameters (Å^2^)

**Table:**

	*U*^11^	*U*^22^	*U*^33^	*U*^12^	*U*^13^	*U*^23^
C1	0.0355 (18)	0.044 (2)	0.046 (2)	−0.0020 (15)	0.0098 (15)	0.0026 (16)
C2	0.0404 (19)	0.0397 (19)	0.0410 (19)	0.0043 (15)	0.0037 (15)	0.0042 (15)
C3	0.0395 (19)	0.0380 (18)	0.0401 (18)	−0.0068 (15)	0.0095 (15)	−0.0047 (15)
C4	0.043 (2)	0.0402 (19)	0.0315 (17)	−0.0016 (15)	0.0028 (14)	−0.0028 (14)
C5	0.054 (3)	0.079 (3)	0.056 (3)	0.010 (2)	−0.007 (2)	0.004 (2)
Cl1	0.0500 (6)	0.0457 (6)	0.0378 (5)	0.0080 (4)	0.0094 (4)	−0.0044 (4)
N1	0.0481 (18)	0.0298 (14)	0.0415 (16)	0.0004 (13)	0.0130 (13)	0.0016 (12)
N2	0.0422 (16)	0.0319 (15)	0.0329 (15)	−0.0012 (12)	0.0103 (12)	0.0013 (12)
O1	0.089 (3)	0.149 (4)	0.080 (3)	−0.040 (3)	0.034 (2)	−0.028 (3)
O2	0.196 (7)	0.199 (7)	0.067 (3)	−0.086 (5)	0.017 (3)	0.048 (4)
O3	0.072 (2)	0.076 (2)	0.074 (2)	0.0078 (18)	−0.0083 (19)	−0.0251 (19)
O4	0.132 (5)	0.151 (5)	0.318 (11)	0.089 (4)	−0.105 (6)	−0.155 (7)

### Geometric parameters (Å, °)

**Table:**

C1—N2	1.485 (5)	C4—C5	1.507 (6)
C1—C2	1.514 (5)	C4—H4A	0.9800
C1—H1B	0.9700	C5—H5A	0.9600
C1—H1A	0.9700	C5—H5B	0.9600
C2—N1	1.465 (5)	C5—H5C	0.9600
C2—H2A	0.9700	Cl1—O4	1.363 (5)
C2—H2B	0.9700	Cl1—O2	1.377 (5)
C3—N1	1.459 (5)	Cl1—O3	1.426 (4)
C3—C4	1.526 (5)	Cl1—O1	1.436 (4)
C3—H3A	0.9700	N1—H1C	0.8998
C3—H3B	0.9700	N2—H2D	0.9000
C4—N2	1.500 (5)	N2—H2C	0.9000
			
N2—C1—C2	109.3 (3)	C3—C4—H4A	108.5
N2—C1—H1B	109.8	C4—C5—H5A	109.5
C2—C1—H1B	109.8	C4—C5—H5B	109.5
N2—C1—H1A	109.8	H5A—C5—H5B	109.5
C2—C1—H1A	109.8	C4—C5—H5C	109.5
H1B—C1—H1A	108.3	H5A—C5—H5C	109.5
N1—C2—C1	113.3 (3)	H5B—C5—H5C	109.5
N1—C2—H2A	108.9	O4—Cl1—O2	111.5 (6)
C1—C2—H2A	108.9	O4—Cl1—O3	112.1 (3)
N1—C2—H2B	108.9	O2—Cl1—O3	110.9 (3)
C1—C2—H2B	108.9	O4—Cl1—O1	107.8 (5)
H2A—C2—H2B	107.7	O2—Cl1—O1	103.9 (4)
N1—C3—C4	114.3 (3)	O3—Cl1—O1	110.3 (2)
N1—C3—H3A	108.7	C3—N1—C2	111.6 (3)
C4—C3—H3A	108.7	C3—N1—H1C	109.0
N1—C3—H3B	108.7	C2—N1—H1C	109.1
C4—C3—H3B	108.7	C1—N2—C4	112.5 (3)
H3A—C3—H3B	107.6	C1—N2—H2D	109.1
N2—C4—C5	110.5 (3)	C4—N2—H2D	109.1
N2—C4—C3	107.8 (3)	C1—N2—H2C	109.1
C5—C4—C3	113.0 (4)	C4—N2—H2C	109.1
N2—C4—H4A	108.5	H2D—N2—H2C	107.8
C5—C4—H4A	108.5		
			
N2—C1—C2—N1	54.6 (4)	C1—C2—N1—C3	−52.3 (4)
N1—C3—C4—N2	−54.1 (4)	C2—C1—N2—C4	−57.7 (4)
N1—C3—C4—C5	−176.4 (3)	C5—C4—N2—C1	−179.4 (3)
C4—C3—N1—C2	52.7 (4)	C3—C4—N2—C1	56.8 (4)

### Hydrogen-bond geometry (Å, °)

**Table:**

*D*—H···*A*	*D*—H	H···*A*	*D*···*A*	*D*—H···*A*
N1—H1C···O1	0.90	2.38	3.258 (6)	166
N1—H1C···O3	0.90	2.54	3.250 (5)	136
N2—H2D···O2^i^	0.90	2.43	2.998 (7)	121
N2—H2C···N1^ii^	0.90	1.99	2.883 (4)	169

Symmetry codes: (i) −*x*+1, −*y*+1, −*z*+1; (ii) −*x*+1/2, *y*+1/2, −*z*+3/2.
